# Oxidative Stress and Cardiometabolic Disorders

**DOI:** 10.1155/2021/9872109

**Published:** 2021-11-05

**Authors:** Roland E. Akhigbe, Ayodeji F. Ajayi, Sahu K. Ram

**Affiliations:** ^1^Reproductive Physiology and Bioinformatics Research Unit, Department of Physiology, College of Medicine, Ladoke Akintola University of Technology, Ogbomoso, Oyo State, Nigeria; ^2^Reproductive Biology and Toxicology Research Laboratories, Oasis of Grace Hospital, Osogbo, Osun State, Nigeria; ^3^Department of Chemical Sciences, Kings University, Ode Omu, Osun, Nigeria; ^4^Department of Pharmaceutical Sciences, Assam University (A Central University), Silchar-788011 (AS), India

Cardiometabolic disorders (CMD) are a cluster of metabolic derangements that increases the susceptibility to insulin resistance and type II diabetes mellitus, systemic hypertension, central obesity, and dyslipidaemia [1, 2]. The rise in the prevalence of CMD is a global phenomenon involving developed nations and underdeveloped and developing countries, leading to a double burden of disease in the tropics. CMD is a multifactorial disorder caused by an intricate interaction between genetics and environmental factors, which lead to increased insulin resistance and circulatory free fatty acids (FFA), lipid and glucose dysmetabolism, and elevated levels of adipokines and cytokines [1, 3–7].

Studies have linked OS with incident CMD [8]. The observed dwindling antioxidant level in advanced age has been shown to reduce cardiotolerance [9]. This is accompanied by arterial thickening, atherosclerosis, vascular damage, and remodeling [10, 11]. These contribute to the development of CMD. Indepth knowledge of the impact of OS in the development of CMD will help to identify possible effective treatment modalities to improve cardiometabolic status.

The purpose of this special issue is to illuminate the effect of OS in the etiopathogenesis of CMD and open new management opportunities. This special issue, oxidative stress and cardiometabolic disorders, contains contributions from 34 reputable scientists from 17 different institutions across the globe.

The first article, “Orosomucoid 1 Attenuates Doxorubicin-Induced OS and Apoptosis in Cardiomyocytes via Nrf2 Signaling,” by X. Cheng et al. documents the rescue effect of orosomucid 1 on doxorubicin-induced cardiotoxicity. The authors clearly demonstrated that orosomucid 1, an acute-phase protein, attenuated inflammation and ischemic stroke in an animal model via upregulation of nuclear factor-like 2 (Nrf2) and suppression of heme oxygenase 1 (HO-1) [12]. In addition, there was a reversal of the impact of ORM1 on doxorubicin-induced OS and apoptosis in cardiac muscles when Nrf2 was silenced. Their study lends credence to the use of orosomucid as a therapeutic strategy for doxorubicin-induced cardiotoxicity.

In the second article, “The Free Radical Scavenging and Anti-Isolated Human LDL Oxidation Activities of *Pluchea indica* (L.) Less. Tea Compared to Green Tea (*Camellia sinensis*),” K. Sirichaiwetchakoon et al. challenged isolated human low-density lipoproteins (LDL) with either 2,2′-azobis (2-amidinopropane) dihydrochloride (AAPH), copper, or 3-morpholinosydnonimine hydrochloride (SIN-1) to induce LDL oxidation [13]. Pluchea indica (L.) Less. Tea (PIT) showed antioxidant potential in all test systems and its capacity to mop off peroxynitrite. PIT performed significantly better than the green tea, *Camellia sinensis* tea (CST), in DPPH and peroxynitrite scavenging assays. Although the antioxidant activities of flavonols and polyphenol catechins in CST have reported earlier [14–17], the study of K. Sirichaiwetchakoon et al. opens a new opportunity for a novel nutraceutical.

N. Zhao et al., in the third article, “Role of Oxidation-Dependent CaMKII Activation in the Genesis of Abnormal Action Potentials in Atrial Cardiomyocytes: A Simulation Study,” probed the influence of oxidation-dependent Ca^2+^/calmodulin-dependent protein kinase II (CaMKII) activation in the genesis of abnormal atrial action potentials (AP) [18]. Zhao and his colleagues explored the intrinsic pathophysiology of OS-induced arrhythmia in the atria. They observed that OS triggered early after depolarizations of AP by modifying the dynamics of transmembrane currents and intracellular calcium cycling. OS caused a rise in cytoplasmic calcium ions via enhancement of L-type Ca^2+^ current and calcium release by the sarcoplasmic reticulum. The resultant increases in intracellular calcium level, elevated Na+/Ca2+ exchange current, and reduced repolarization of the action potential. This culminated in prolonged AP and consequent early after depolarizations.

The study in the fourth article “*Qiliqiangxin* Improves Cardiac Function through Regulating Energy Metabolism via HIF-1***α***-Dependent and Independent Mechanisms in Heart Failure Rats after Acute Myocardial Infarction,” authored by Y. Wang et al. was designed to evaluate the influence of Qiliqiangxin, QL, on energy metabolism in experimental myocardial infarction and the role of hypoxia-inducible factor 1*α* (HIF-1*α*) signaling [19]. Acute myocardial infarction (AMI) was established by ligating the left anterior descending coronary artery in adult male Sprague Dawley rats, and animals with an ejection fraction < 50% at two weeks postoperation were considered animals with heart failure. They randomized rats into sham, MI-induced, MI + QL, and MI + QL+2-MeOE2 groups. They found out that QL significantly improved cardiac function and myocardial capillary density, reduced serum NT-proBNP, and attenuated myocardial fibrosis. This was accompanied by enhanced glucose and free fatty acid uptake, glycolysis, and ATP production, as well as upregulation of the protein expression of vascular endothelial growth factor (VEGF), myocardial glucose oxidation enzyme expression, and CD 31 via regulation of HIF-1*α*/VEGF signaling.

The fifth article, “*Dracocephalum moldavica L.* Extracts Protect H9c2 Cardiomyocytes against H2O2-Induced Apoptosis and OS,” evaluated the cardioprotective potential of *Dracocephalum moldavica L*., a phytomedicinal plant used in the management of cardiovascular diseases in China against H_2_O_2_-induced apoptosis and OS in H9c2 cells. M. Jin et al. pretreated H9c2 cells with *Dracocephalum moldavica L*. before challenging with H_2_O_2_ [20]. *Dracocephalum moldavica L*. therapy was found to attenuate H_2_O_2_-induced decline in cell viability, SOD activity, and mitochondrial membrane potential. The phenol- and flavonoid-rich *Dracocephalum moldavica L*. also abrogated H_2_O_2_-induced elevations in ROS generation and concentrations of MDA and lactate dehydrogenase. *Dracocephalum moldavica L*. cardioprotective activities were revealed to be mediated through upregulation of the Bcl-2 expression and downregulation of the Bax and caspase 3 expression.

In the sixth article, “Multimodal *α*-Glucosidase and *α*-Amylase Inhibition and Antioxidant Effect of the Aqueous and Methanol Extracts from the Trunk Bark of *Ceiba pentandra*,” T.B. Nguelefack et al. explored the postprandial modulatory activities and antioxidant potentials of *Ceiba pentandra* aqueous and methanolic stem bark extracts [21]. They demonstrated that the phenol- and flavonoid-rich extracts of *Ceiba pentandra* significantly reduced postprandial hyperglycemia by inhibiting protein oxidation, *α*-amylase, and *α*-glucosidase through scavenging reactive oxygen species. These findings are extensions of their previous studies that revealed that *Ceiba pentandra* promotes glucose utilization and reduces hepatic glucose release [22], upregulates glycogen synthesis, and impairs gluconeogenesis [23], inhibits lipid peroxidation and shows antioxidant activity against DPPH and hydroxyl radical [22], and demonstrated antidiabetic properties in dexamethasone-treated rats [24] and high-fat diet/streptozotocin-treated rats [25].

We hope our readers will find these articles interesting and stimulating. The articles and recommendations of the contributing experts will hopefully spur further discussion and expand research in these biomedical areas.

*Roland E. Akhigbe* *Ayodeji F. Ajayi* *Sahu K. Ram*
